# Human sperm heads harbor modified YsRNA as transgenerationally inherited non-coding RNAs

**DOI:** 10.3389/fgene.2023.1294389

**Published:** 2023-12-13

**Authors:** Darja Elzer, Michelle Bremser, Hans Zischler

**Affiliations:** Division of Anthropology, Faculty of Biology, Institute of Organismic and Molecular Evolution, Johannes Gutenberg University Mainz, Mainz, Germany

**Keywords:** human sperm heads, non-coding RNA payload, 2′-O methylation, transgenerational epigenetic inheritance, YRNA, small YRNA, piRNA

## Abstract

Most epigenetic information is reprogrammed during gametogenesis and early development. However, some epigenetic information persists and can be inherited, a phenomenon that is common in plants. On the other hand, there are increasing examples of epigenetic inheritance in metazoans, especially for small non-coding RNAs. The presence of regulatory important RNAs in oocytes is undisputed, whereas the corresponding RNA payload in spermatozoa and its regulatory influence in the zygote and early embryogenesis is largely enigmatic. For humans, we herein describe small YRNA fragments (YsRNA) as a paternal contribution to the zygote. First, we trace the biogenesis of these YsRNAs from the source YRNAs with respect to the 5′ and 3′ modifications. Both the length and modifications make these YsRNAs reminiscent of canonical piRNAs that are not derived from piRNA clusters. Second, from the early stages of spermatogenesis to maturation in the epididymis, we observe distinct YsRNA profile dynamics in the male germline. We detected YsRNAs exclusively in mature sperm heads, the precursor of the male pronucleus in the zygote, suggesting an important role of the epididymis as a site for transmitting and modification of epigenetic information in the form of YsRNA between soma and germline in humans. Since this YsRNA-based epigenetic mechanism is effective across generations, we wondered whether this phenomenon of epigenetic inheritance has an adaptive value. Full-length YRNAs bind to Ro60, an RNA chaperone that additionally binds to non-coding RNAs. We described the profiles of non-coding RNAs bound to Ro60 in the human sperm head and detected specific binding profiles of RNA to Ro60 but no YRNA bound to Ro60. We hypothesize that the sperm head Ro60 system is functional. An adaptive phenotype mediated by the presence of a large amount of YsRNA in the sperm head, and thus as a paternal contribution in the zygote, might be related to an association of YsRNA with YRNA that prevents the adoption of a YRNA secondary structure capable of binding to Ro60. We hypothesize that preventing YRNAs from acting as Ro60-associated gatekeepers for misfolded RNAs in the zygote and early development may enhance RNA chaperoning and, thus, represent the adaptive molecular phenotype.

## 1 Introduction

Spermatogenesis is a dynamic developmental process starting from stem cell proliferation and differentiation, meiotic cell divisions, and finally, an almost complete replacement of the canonical histones by protamines in the sperm head, leading to a stronger genome compaction. The main task during this process is to realize different genome safeguarding mechanisms and to undergo a differentiation program characterized by an extreme dynamic of the stage-specific transcriptomes, leading to a highly specialized sperm cell with markedly reduced transcriptional activity (reviewed in Lesch B.J. 2020). The extreme complexity of the transcriptome during spermatogenesis is characterized by the fact that nearly the whole genome is expressed in testes, more than in any other cell (Soumillon et al., 2013). This phenomenon is speculated to be linked to an evolutionary important “transcriptional scanning” of the genome exploiting the mechanism of the transcription-coupled repair machinery (Xia et al., 2020). In mammals, messenger RNA (mRNA) is massively eliminated during late spermiogenesis (Gou et al., 2014) and small RNAs are eliminated during post-testicular maturation of sperms. Thus, PIWI-interacting RNAs (piRNAs) are almost completely absent from ejaculated sperms, in which a small amount of small non-coding RNAs (sncRNAs) composed primarily of transfer RNA (tRNA) cleavage fragments together with a smaller population of microRNAs (Peng et al., 2012) can be traced. Hence, the characteristic feature of the germline epigenome erasure/re-establishment also affects the non-coding RNA (ncRNA) transcriptome in the male germline. Summarizing current research in metazoans, it is widely accepted that the oocyte carries the majority of relevant regulator RNAs that function in the early embryo of most species. However, there is substantial and increasing evidence that sperms also carry a functional RNA payload (Bošković and Rando, 2018). Theoretically, small RNAs in the male germline show potential to affect subsequent generations either indirectly by directing chromatin or DNA modifications during gametogenesis or directly via delivery to the zygote. Since the sperm nucleus, located in the sperm head, is the precursor of the male pronucleus (Lassalle and Testart, 1991), sperm head-located ncRNAs could influence the transcriptomes of early zygotes and before the zygotic genome activation (ZGA) becomes efficient.

It is, therefore, of utmost importance to precisely describe the complete RNA cargo of mature male gametes, its biogenesis with respect to possible modifications, and thus functionality and the RNA profiles during development from testis and early stages to ejaculated sperms. In addition, it is important to obtain experimental evidence for the possible functions of small RNAs in the early embryo (Bošković and Rando, 2018). This phenomenon of epigenetic inheritance has been demonstrated for several model organisms including domestic animals (for review, check Zhang and Sirard, 2021). We herein focus on the human situation and the small RNA payload of mature sperm heads.

An annotation of human sperm head small RNA by UNITAS (Gebert et al., 2017) revealed that a major fraction of miscellaneous RNA (miscRNA) and RNA that cannot be annotated are derived from YRNAs. YRNAs were originally found in the cytoplasm of cells and therefore given the prefix “Y” (Lerner et al., 1981). There are four YRNA loci in humans, hY1 with a size of 112 nt, hY3 with a size of 101 nt, hY4 with 93 nt in size, and hY5 with a size of 83 nt (Gulìa et al., 2020), which are clustered on chromosome 7q36 (Maraia et al., 1994; Maraia et al., 1996). The 5′ end, having a triphosphate, and 3′ end typically hybridize to form a double-stranded stem domain divided into a lower and upper stem domain (Kowalski and Krude, 2015). YRNAs are RNA-polymerase III (Pol III)-transcribed, and the La-binding site is located at the 3′ polyuridine tail. The La protein is a phosphoprotein that associates with newly synthesized RNA Pol III transcripts, including pre-tRNAs and YRNAs (Wolin and Cedervall, 2002). The binding of the La protein to this tail protects the YRNA from 3′ and 5′ exonucleolytic degradation and promotes its retention in the nucleus (Simons et al., 1996; Wolin and Cedervall, 2002). The upper stem domain is important for chromosomal DNA replication, and the lower stem domain has a Ro60-binding site and is, when bound to Ro60, involved in RNA stability and stress response (Kowalski and Krude, 2015). YRNAs not only exist in their full length but also in fragments between 25 nt and 35 nt (Röther and Meister, 2011), which are referred to as YsRNAs. Interestingly, the range of the YsRNA fragment length falls together with RNase L-cleavage sites in the full-length YRNAs (Donovan et al., 2017; Figure 2B). To detect the small RNA profiles, we used small RNA sequencing, mapping tools, and local BLAST analyses with the YRNA homologs as the database. To elucidate the YsRNA profiles during spermatogenesis, NGS sequencing was performed for one human testis small RNA sample and sperm head samples from six individuals. In addition, we downloaded small RNA datasets from different early stages of human spermatogenesis, human oocytes, semen vesicles, and epididymis from the NCBI Sequence Read Archive (SRA), the latter to take reproductive support tissue into account. To detect small RNA modifications that were introduced during the biogenesis of YsRNAs, we carried out T4 polynucleotide kinase (PNK)-, RNA 5′ pyrophosphohydrolase (RppH)-, and before/after oxidation experiments with sperm head and testis small RNA by comparatively quantifying the small RNA-seq readouts with respect to the various YsRNAs.

## 2 Materials and methods

### 2.1 Sperm head preparation

Semen samples were collected from six volunteers between the ages of 24 and 62. Donors were asked to abstain from sexual activity for 2–3 days. The entire ejaculates gained by masturbation were collected in a sterile 50-mL tube. The samples were used within 1 hour after ejaculation. The semen samples were evenly distributed to 2-mL reaction tubes and centrifuged at room temperature at 16,000 × g for 5 min. The supernatants were discarded, and the pellets were resuspended in 700 µL TEN [20 mM TRIS, 20 mM EDTA, 200 mM NaCl, (pH 8.0)], 400 µL HPLC gradient grade water, and 300 µL 10% SDS, and centrifuged at room temperature at 16,000 × g for 5 min. The supernatants were removed, and the washing steps were repeated a second time. After the final wash, the pellets were dissolved in 48 µL ddH_2_O and 2 µL 1 M dithiothreitol (DTT) or stored at −80°C until further usage.

### 2.2 RNA isolation

Total RNA was isolated using the Quick-RNA™ MiniPrep kit from Zymo Research, according to the manufacturer’s instructions, whereby the samples were mixed with 600 µL of RNA lysis buffer in the first step. The isolated RNA was dissolved in 20 µL RNase-free water and stored at −80°C until further usage.

### 2.3 Small RNA sequencing

The isolated sperm heads and testis RNA samples were sent to BGI for library preparation and small RNA sequencing. RNA samples from the same donor sperm heads were pooled from several preparations and combined to meet the company’s requirements of a total amount exceeding 1 µg RNA. Size fractions with a cut-off at 50 nt were used to enrich small RNA for library preparation, and the sequencing strategy was SE 50.

### 2.4 Oxidation and ß-elimination of the sperm head and testis RNA

Periodate treatment of RNA coupled with subsequent ß-elimination was performed essentially, as described by Williams et al. (2015). Each 2 µg of human testis RNA (BioChain^®^; #R1234260-50) and total RNA isolated from sperm heads were dried and afterward resuspended in 17.5 µL borate buffer [4.38 mM Na_2_B_4_O_7_*10H_2_O and 50 mM H_3_BO_3_ (ph 8.6)]. A volume of 7.5 µL 100 mM NaIO_4_ was added, and the samples were incubated for 10 min in the dark at 24°C. A volume of 2 µL HPLC water and 3 µL 50% glycerol were then added to the samples to quench the reaction with glycerol at a final concentration of 5%. To this end, the samples were incubated for another 10 min in the dark at 24°C. Afterward, the samples were concentrated to 5 µL in a SpeedVac. For subsequent ß-elimination, 50 µL borate buffer (33.75 mM Na_2_B_4_O_7_*10H_2_O and 50 mM H_3_BO_3_ [pH 9.5, adjusted by NaOH]) was added to the samples, followed by a 90-min incubation at 45°C. Samples were then precipitated, by adding 150 µL 100% ethanol together with 2 µL glycogen as the carrier. Precipitation was carried out overnight at −20°C. After centrifugation, the pellets were dried and afterward resuspended in 20 µL HPLC water. For the control samples, 2 µg of testis and sperm head RNA were mixed with HPLC water to reach a volume of 20 µL. Small RNA-Seq was done by BGI implementing the abovementioned strategies with respect to size fractionation and the sequencing strategy. Unprotected RNAs with vicinal 2′ and 3′ diol groups react with sodium periodate (NaIO_4_). During subsequent ß-elimination, these RNAs are shortened by 1 nt, leaving them with a 3′ monophosphate. This 3′ monophosphate prevents adapter ligation during library preparation.

### 2.5 PNK and RppH treatment

The YRNA source genes of the respective YsRNAs are triphosphorylated at the 5′ terminus, a modification which would prevent them from ligation to adapters and thus representation in NGS readouts. To find out whether the sperm head YsRNAs have 5′ and 3′ modifications and whether the thus modified YsRNAs can be enriched in NGS analyses after enzymatic restoration of the canonical termini, an approach for enzymatic manipulation of the 5′ and 3′ termini was performed, which was essentially described by Shi et al. (2021). To this end, T4 polynucleotide kinase (PNK, 10U/µl/NEB) and RNA 5′ pyrophosphohydrolase (RppH, 5U/µl/NEB) were used individually and in combination to target 5′ and 3′ modifications. T4 PNK catalyzes the transfer of phosphate from ATP to the 5′ -hydroxyl terminus of polynucleotides and catalyzes 5′-P dephosphorylation and exchange reactions as well (Eun, 1996). It also catalyzes the removal of 3′-phosphoryl groups. RNA 5′ pyrophosphohydrolase removes pyrophosphate from the 5′ end of triphosphorylated RNA, leaving a 5′ monophosphate RNA. Samples from one individual were collected, and sperm heads were isolated and pooled. RNA was isolated, dissolved, and divided into four fractions. The untreated sample contained 1.203 µg RNA, and the PNK, RppH, and PNK+RppH samples contained 1.508 µg RNA as the starting material, respectively. For the treatment with PNK, the RNA sample was mixed with 2 µL T4 PNK buffer and 2 µL PNK and HPLC water to reach a final volume of 20 µL. The sample was incubated at 37°C for 30 min. For the RppH treatment, the sample was mixed with 2 µL NEB buffer, and 2 µL RppH and HPLC water to reach a final volume of 20 µL. The sample was incubated at 37°C for 30 min. For the combined treatment sample, PNK treatment was done, followed directly by RppH treatment as mentioned above. After the incubation time, all samples were ethanol-precipitated for 30 min at −20°C. After centrifugation, the pellets were dried and resuspended in 15 µL HPLC water. Small RNA-Seq was performed by BGI as outlined above.

### 2.6 RNA immunoprecipitation

To investigate further the already-known function of YRNAs, we concentrated on their binding capability to Ro60 and the possible role of YsRNA in this context. To do so, one sample of isolated sperm heads was resuspended in 1 mL PBS, cross-linked using formaldehyde at a final concentration of 1%, and incubated for 10 min at room temperature. A volume of 265 µL ice-cold 1 M glycine was added, and the sample was incubated on a shaker for 5 min at room temperature, and centrifuged at 13,000 × g and 4°C for 5 min. The pellet was washed three times with 1.5 mL ice-cold PBS and centrifuged at 13,000 × g for 5 min at 4°C. After the final wash, the pellet was dissolved in 500 µL cold RIPA buffer [50 mM TRIS-HCl, 150 mM NaCl, 2 mM EDTA (pH 8), 1% Triton X-100, 0.5% sodium deoxycholate, and 0.1% SDS] and incubated for 10 min on ice. To open the sperm heads, the sample was sonicated using the Covaris E220 Focused-Ultrasonicator. The peak incident power was set to 140, duty factor to 10%, cycle of burst to 200, temperature to 20°C, and duration to 120 s, after the sonication immunoprecipitation was performed. To do so, 20 µL Protein-A/G beads were washed twice with 1 mL 1x PBS + 0.1% Triton X-100. The supernatant was discarded, and the beads were resuspended in the 1 mL blocking solution [1 mM EDTA, 10 mM TRIS-HCl (pH 8), 1% BSA, and 1% polyvinylpyrrolidone] and incubated for 15 min on a rotor at room temperature. The beads were placed in a magnetic rack, and the supernatant was discarded. A total of 5 µL anti-TROVE2-antibody (antibodies-online; ABIN7188011) was dissolved in 1 mL 1x PBS + 0.1% Triton X-100, mixed with the beads and incubated on a rotor for 30 min at room temperature. The tube was placed in a magnetic rack, and the supernatant was discarded. The beads were washed twice with 1 mL 1x PBS + 0.1% Triton X-100 and afterward dissolved in 100 µL 1x PBS + 0.1% Triton X-100. The sample was mixed with the beads and incubated on a rotor overnight at 4°C, placed in a magnetic rack to remove the supernatant. The beads were washed twice with low salt buffer [2 mM EDTA, 20 mM TRIS-HCl (pH 8), 150 mM NaCl, 1% Triton X-100, and 0.1% SDS], followed by two wash steps with high salt buffer [2 mM EDTA, 20 mM TRIS-HCl (pH 8), 500 mM NaCl, 1% Triton X-100, and 0.1% SDS] and a final wash with LiCl buffer [1 mM EDTA, 10 mM TRIS-HCl (pH 8), 0.25 M LiCl, 1% Triton X-100, and 1% sodium deoxycholate]. The supernatant was discarded. To remove cross-links, the sample was mixed with 120 µL elution buffer (100 mM NaHCO_3_, 1% SDS), incubated for 15 min at 30°C, and centrifuged at 2,000 × g for 1 min at room temperature. The supernatant was put in a fresh reaction tube, and 4.8 µL 5 M NaCl and 2 µL Proteinase K (20 mg/mL) were added. The sample was incubated for 30 min at 65°C, 400 µL TRIzol was added, and the sample was incubated for 5 min at room temperature. After adding 80 µL chloroform and incubating for 3 min at room temperature with intermitted vortexing, the sample was centrifuged at 16,000 × g for 5 min at 4°C. The aqueous phase was transferred into a fresh reaction tube and precipitated by adding 750 µL ice-cold ethanol, 1 µL glycogen, and 25 µL NaAc, and was incubated at −20°C overnight. After centrifugation at 7,500 × g for 1 h at 4°C, the pellet was shortly air-dried and dissolved in 40 µL RNase-free water and incubated for 15 min at 60°C. Custom library preparation and sequencing were done by Novogene—to enrich for lincRNA—rRNA removal was done, and library was prepared without size selection.

### 2.7 Bioinformatics analysis

#### 2.7.1 UNITAS annotation

To analyze the data provided by BGI, UNITAS 1.8.0 was used to get a classification into different small RNA classes. Sequences annotated to either miscRNA or sequences that could not be annotated were retrieved as collapsed FASTA files, aligned with SEAVIEW, and used to query the non-redundant database from the NCBI.

#### 2.7.2 Local BLAST of transcriptomic data with YRNA sequences

FASTQ files of small RNA transcriptomes or transcriptomes, as retrieved from the SRA (Vojtech et al., 2014; Yang et al., 2019; Gong et al., 2022), were adapter-trimmed and mapped to the human genome (hg38) using Trim Galore and HISAT2. Next, we ran local BLAST analyses of the transcriptome data with YRNA sequences as the database. Sequences of the four YRNA homologs were retrieved from the NCBI. To take the YRNA secondary structure into account and minimize redundant hits due to the intrinsic stem part reverse complementarity of YRNAs, the sequences were split into two halves (5′ and 3′). BLASTN settings included “-perc_identity 100” to retrieve exclusively 100% identical hits. Hits were corrected for the overall alignment rate in the respective datasets, as determined by HISAT2, and given in reads per million of alignable sequences. Two-way ANOVA tests, including Tukey’s multiple comparison test, using the GraphPad Prism version 10.0.2 for Windows (GraphPad Software, Boston, Massachusetts USA, www.graphpad.com), were used for statistical analyses of the data.

#### 2.7.3 Bioinformatics evaluation of RIP data

FASTQ files, as obtained from Novogene, were HISAT2-mapped to hg38, and the resulting BAM files were used to extract the RNA-seq reads defined by both the hg38 transcriptome ENSEMBL GTF file and GTF, as obtained from the LNCipedia database version 5.2 (Volders et al., 2019). Finally, the counting of mapped reads was carried out by applying the FeatureCounts routine from the Subread package (Liao et al., 2014). The counts were corrected for the alignable reads in the dataset, as obtained by HISAT2, and are given in reads per million alignable sequences.

## 3 Results

### 3.1 Small non-coding RNA-Seq and UNITAS annotation uncover fragments of YRNA as a major RNA payload in sperms

Sperm heads were obtained from six individual ejaculates, RNA isolated, and small RNA-sequenced. A range of 25–46 million total reads were obtained from each individual, and the quality of the datasets was determined by HISAT2 mapping to hg38, which resulted in a mean percentage of alignable reads of 80.49%. A complete annotation of the sperm head small RNA payload was done by UNITAS. As a result, the no annotation fraction values ranged from 40% to 60%, and mean values for rRNA, tRNA, miRNA, and lincRNA were 19.06%, 9.76%, 5.15%, and 2.53%, respectively. Other fractions mapped to piRNA clusters (5.49%) and miscRNA (4.07%), respectively (for the respective individual donut charts, as obtained by UNITAS, see Supplementary Figure S1, and for the individual values, see Supplementary Table S2). To get an idea about the not-annotated and miscRNA sequences, the respective collapsed FASTA files provided by UNITAS were retrieved and aligned by SEAVIEW. BLAST searches of consensus queries revealed that the main fractions of these sequences were similar to human YRNAs.

To more precisely describe these short YRNA sequences with respect to their YRNA homologs and to narrow down the regions of similarity, local BLAST searches were carried out. To this end, the four human homologs of YRNAs (hY1, hY3, hY4, and hY5)—each approximately 100 nt in length—were divided into roughly two halves and used as the database, to avoid confounding of our results due to the cryptic complementarity of the YRNA stem regions.

### 3.2 The 5′ region of hY1 is enriched in human sperm heads and almost absent in oocytes

**FIGURE 1:**
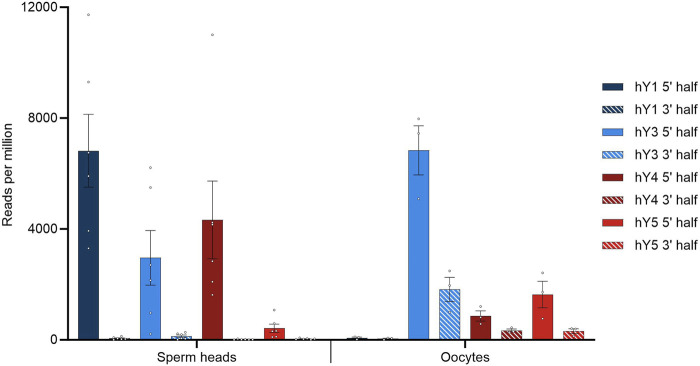
BLASTN results querying small RNA with YRNA as the database. Hits were corrected for the overall alignment rate in the respective datasets, as determined by HISAT2, and given in reads per million of alignable sequences. Dots indicate individual hit numbers, and bars indicate the standard error of mean (SEM).

Our sperm head small RNA data were blasted against the YRNA database. Only hits with a percentage identity of 100% were used to calculate mean values for YRNA hits. The SRA-deposited oocyte datasets (BioProject PRJNA376426) were quality-checked with TRIM GALORE, and clean sequences were retrieved. Subsequently, HISAT2 was carried out to determine the overall alignment rate. The clean sequences were used for the local BLAST analyses. The comparison of perfect hit numbers specific for the 5′ and 3′ parts of the YRNA sequences and separated for the four homologous YRNA loci is depicted for both sperm heads and oocytes, as shown in Figure 1.

In general, the quantitative distribution of YsRNA uncovers that the 5′ regions of the YRNAs can be detected in high abundance in all datasets, whereas the respective 3′ parts of the YRNAs are virtually absent in sperm heads. This 5′ part to 3′ part imbalance is less pronounced in oocytes, however, with a clear tendency of preponderance of 5′ YRNA regions as well. Interestingly, the 5′ part of hY1 is enriched in human sperm heads and is almost absent in oocytes; on account of this, the 5′ fragments derived from hY3 show the highest abundance.

### 3.3 Sperm head hY1 5′ regions are predominantly 30 nt and 31 nt in length

Next, we asked if the YsRNAs detected exhibit a continuous size profile or if discrete size classes could be uncovered. To this end, we counted the number of perfect hits with a certain length (see Supplementary Figure S2).

The length distributions of Ys1RNA (5′) and Ys3RNA (5′) peak at 30 nt and 31 nt, respectively, starting from the first and second bases of the source YRNA. This length falls into the size range of piRNAs. Ys1RNAs of these sizes are restricted to the sperm heads, whereas these YRNA fragments are virtually absent in oocytes. The most abundant YsRNA length in oocytes is 31 nt and 32 nt (data not shown). As the precursor of the male pronucleus, 5′ Ys1RNA, in the sperm head, thus, constitutes a paternal contribution to the zygote.

### 3.4 5′ and 3′ modifications of YsRNA point toward YRNA as source genes for piRNAs in human sperm heads

**FIGURE 2:**
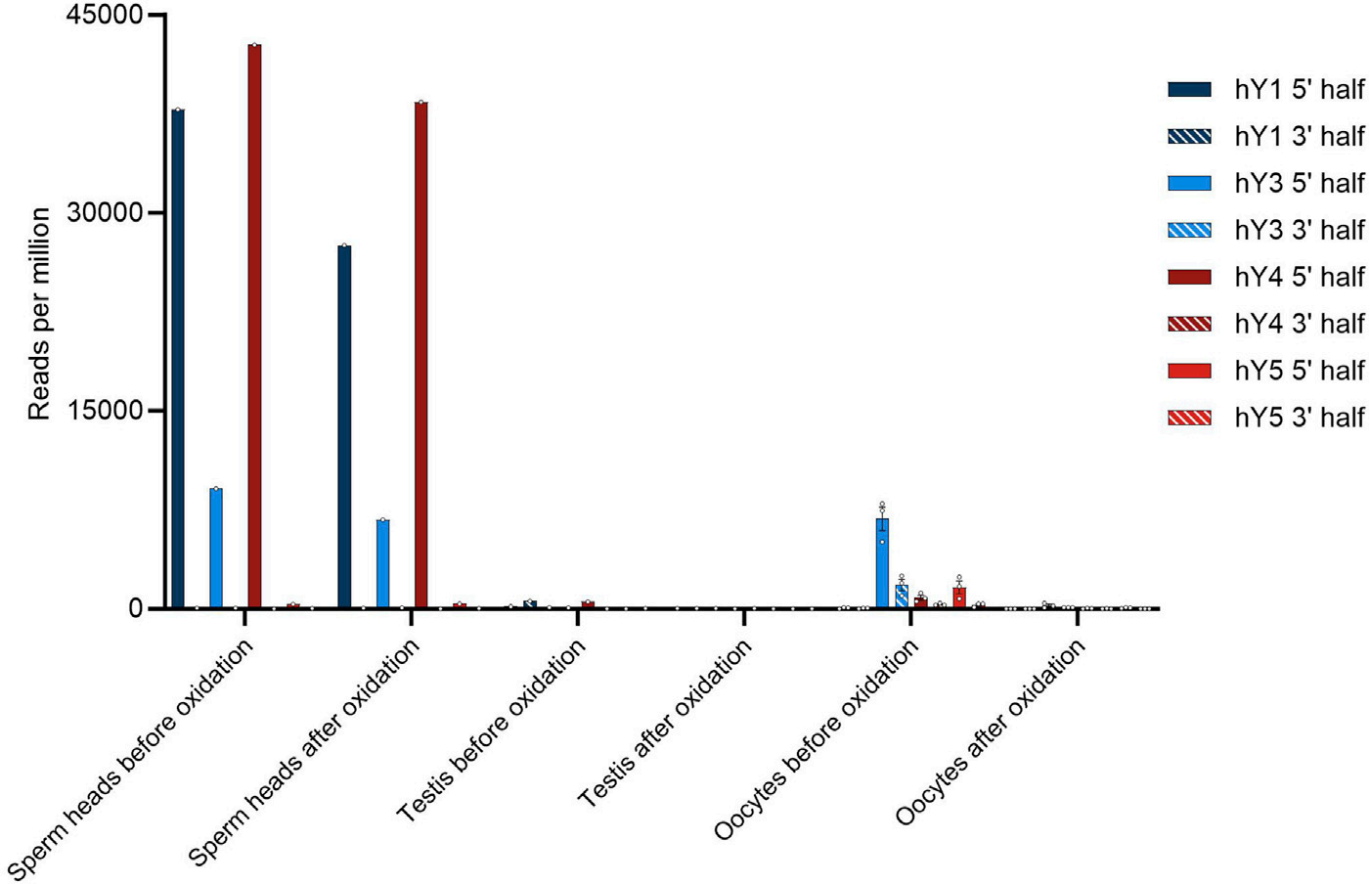
Number of YsRNA hits in sperm heads, testis, and oocytes before and after oxidation given in reads per million alignable reads. Individual hits are shown as dots with the error bars (SEM).

To investigate further the similarity of YsRNA and piRNA, we checked sperm head RNA for possible modifications that are typical for piRNAs by setting up an oxidation/ß-elimination experiment. To check with the situation in the early stages of spermatogenesis and to have an internal control, we included bulk testis RNA in our assay. Samples from the same donor were used to find out if YsRNAs are protected at their 3′ end due to 2′-O-methylation. To this end, periodate treatment and ß-elimination were performed. Small RNA-Seq was performed, and the respective data were blasted against the YRNA database. Early stages of spermatogenesis were represented by treating human testis RNA, as described. In addition, oocyte data from before/after oxidation setups were included in our analysis to directly compare the situation in early stages of spermatogenesis with sperm heads and the maternal site. Figure 2 summarizes the results of the ß-elimination experiments.

In the sperm head, the Ys1RNA patterns reveal extensive protection of these RNAs—more precisely the 31-nt fraction (see Supplementary Figure S3). Both the fragment size and 3′ modifications make these fragments reminiscent of canonical piRNA, albeit not derived from piRNA clusters. Sperm heads, in addition, contain considerable amounts of Ys4RNA that are protected at their 3′ end due to 2′-O-methylation. In contrast, these piRNA-like YsRNAs are virtually absent from early developmental stages of spermatozoa, an observation we confirmed by analyzing bulk RNA from testis, containing spermatogonia, spermatocytes, spermatids, and spermatozoa of different developmental stages. This makes the sperm head YsRNA/piRNAs almost entirely an exclusive RNA payload and paternal contribution to the zygote transmitted via the male pronucleus.

The source genes of the YsRNA are 5′ triphosphorylated, a modification that would prevent a representation in NGS libraries. To test if this 5′ modification is present in YsRNA and to elucidate further modifications, we set up a scheme of tests, similar to the PANDORA-Seq-strategy (Shi et al., 2021). Thus, RppH and PNK treatment was carried out in a separate and combined setting. NGS readouts were HISAT2-mapped to hg38 to see if the different treatments resulted in a significantly lower alignment rate, indicating the introduction of inadvertent chemical modifications preventing adapter ligation. Focusing on the canonical 5′ triphosphate of YRNA source gene products, we could observe an RppH effect in 5′ Ys4RNA and 5′ Ys5RNA, representing an internal positive control and indicating the presence of 5′ triphosphates in these molecules. For Ys1RNA and Ys3RNA, the untreated sample results in the highest abundance of the fragments, which is indicative that these fragments possess canonical 5′ phosphates that can be ligated and sequenced.

Upon treating the samples with PNK alone or in combination with RppH, the hit numbers for the 5′ regions of both hY1 and hY4 are markedly reduced.

**FIGURE 3:**
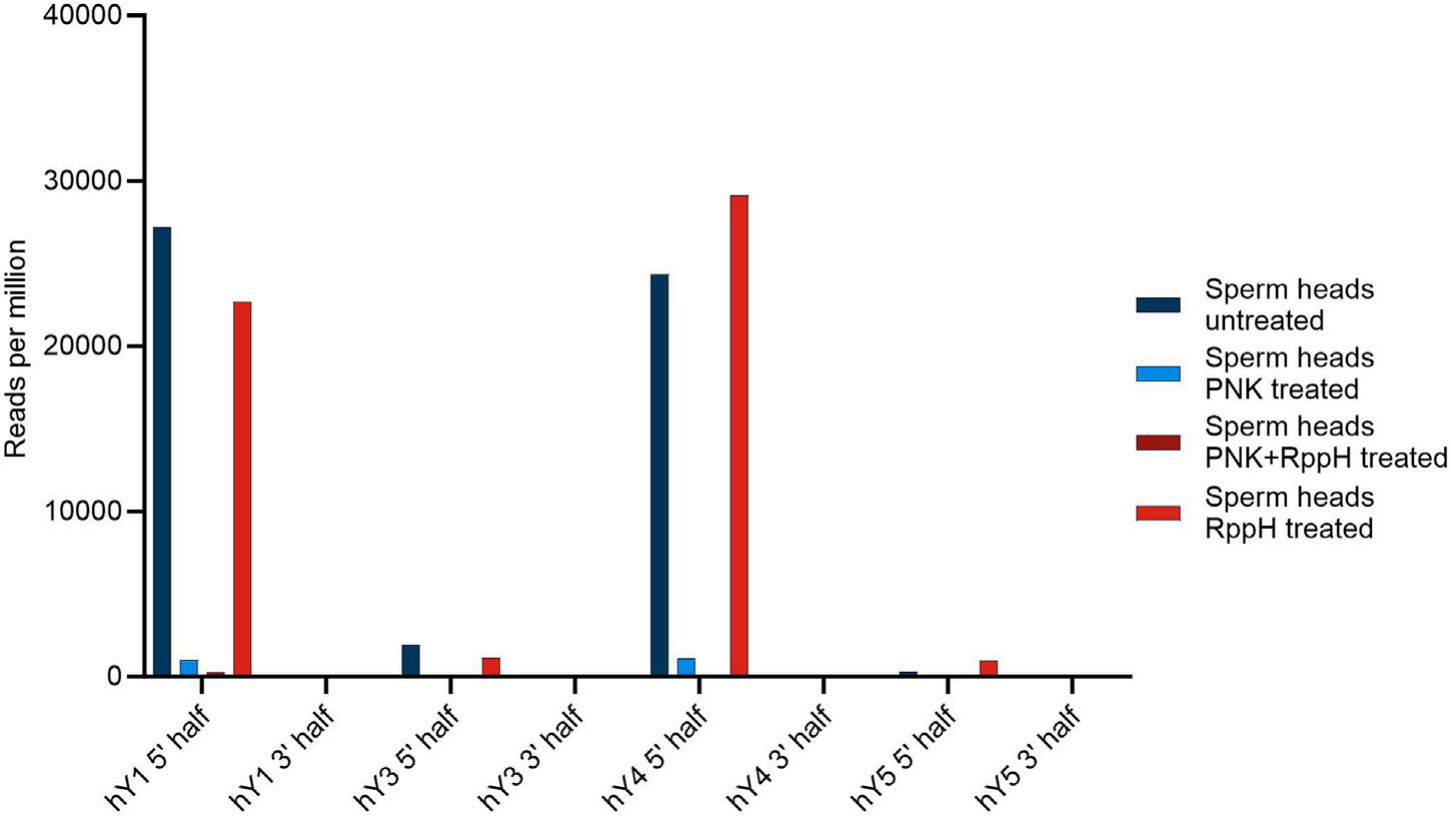
Number of YsRNA hits in sperm heads without treatment, treatment with PNK, RppH, and both enzymes, respectively, in reads per million alignable reads shown for the four YRNAs, divided in a 5′ half and a 3′ half.

An overview of the results is shown in Figure 3.

### 3.5 YsRNAs are soma-germline-transmitted presumably via epididymal vesicles

**FIGURE 4:**
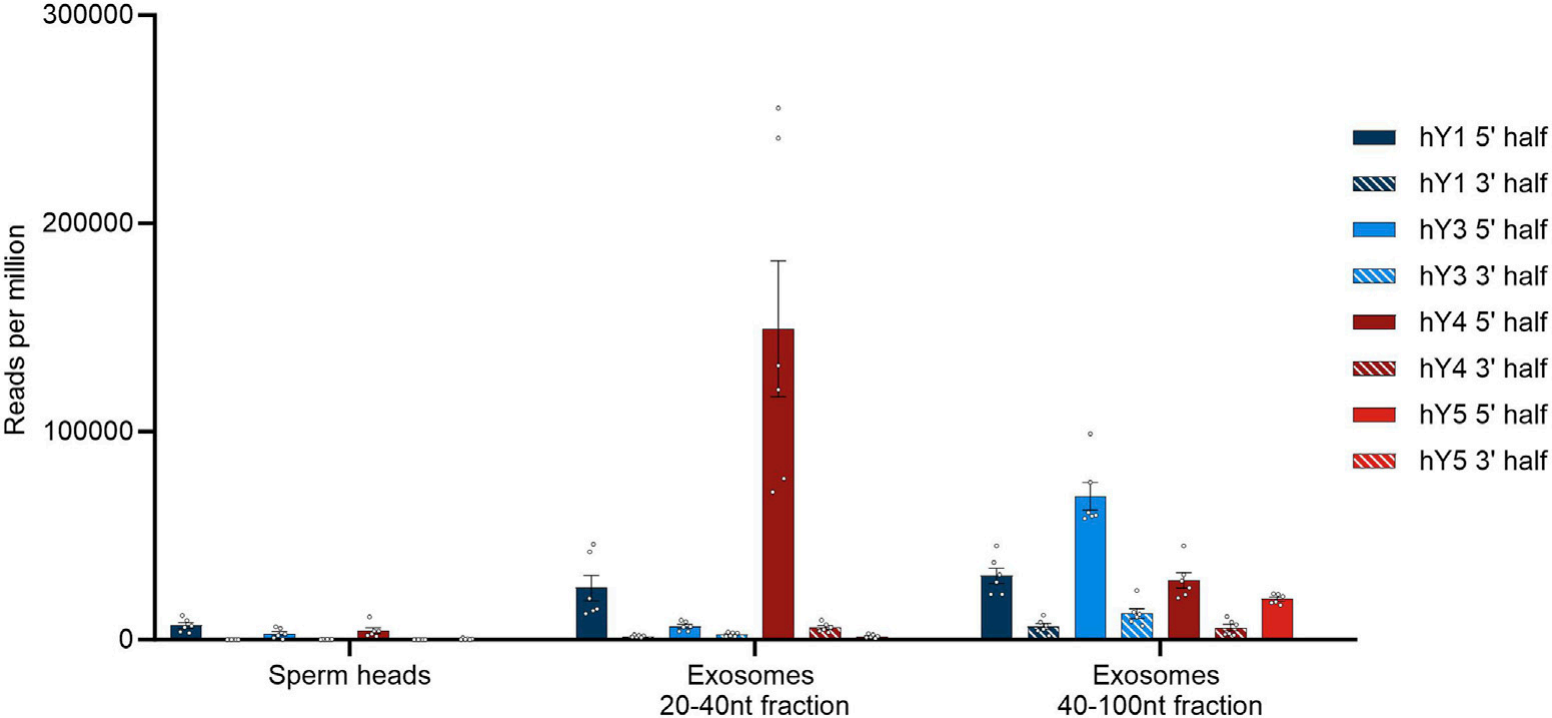
YsRNA hits shown for sperm heads and exosomes in reads per million mappable reads for the four YRNA homologs divided in a 5′ half and a 3′ half. Bars are shown with error bars (SEM), and individual hits are shown as dots.

We could not observe biogenesis of the YsRNA/piRNA profiles along with the developmental stages of sperm cells, instead witnessing a sudden appearance in mature sperms. Interestingly, YRNAs and YsRNAs were described to be highly abundant in seminal plasma exosomes, more precisely in two size ranges from 20 to 40 nt and 40 to 100 nt (Vojtech et al., 2014). We downloaded the respective SRA accessions (BioProject PRJNA242348) and analyzed the data with respect to the abundances of the different YsRNAs. The same BLAST strategies, as above, were applied, and a comparison of sperm heads and exosomes with respect to hits per million of mappable reads is given in Figure 4.

Seminal plasma exosomes do contain a considerable fraction of YsRNAs that can be found in human sperm heads, albeit the profiles exhibiting quantitative differences mainly in the Ys1RNA to Ys3RNA ratio.

**FIGURE 5:**
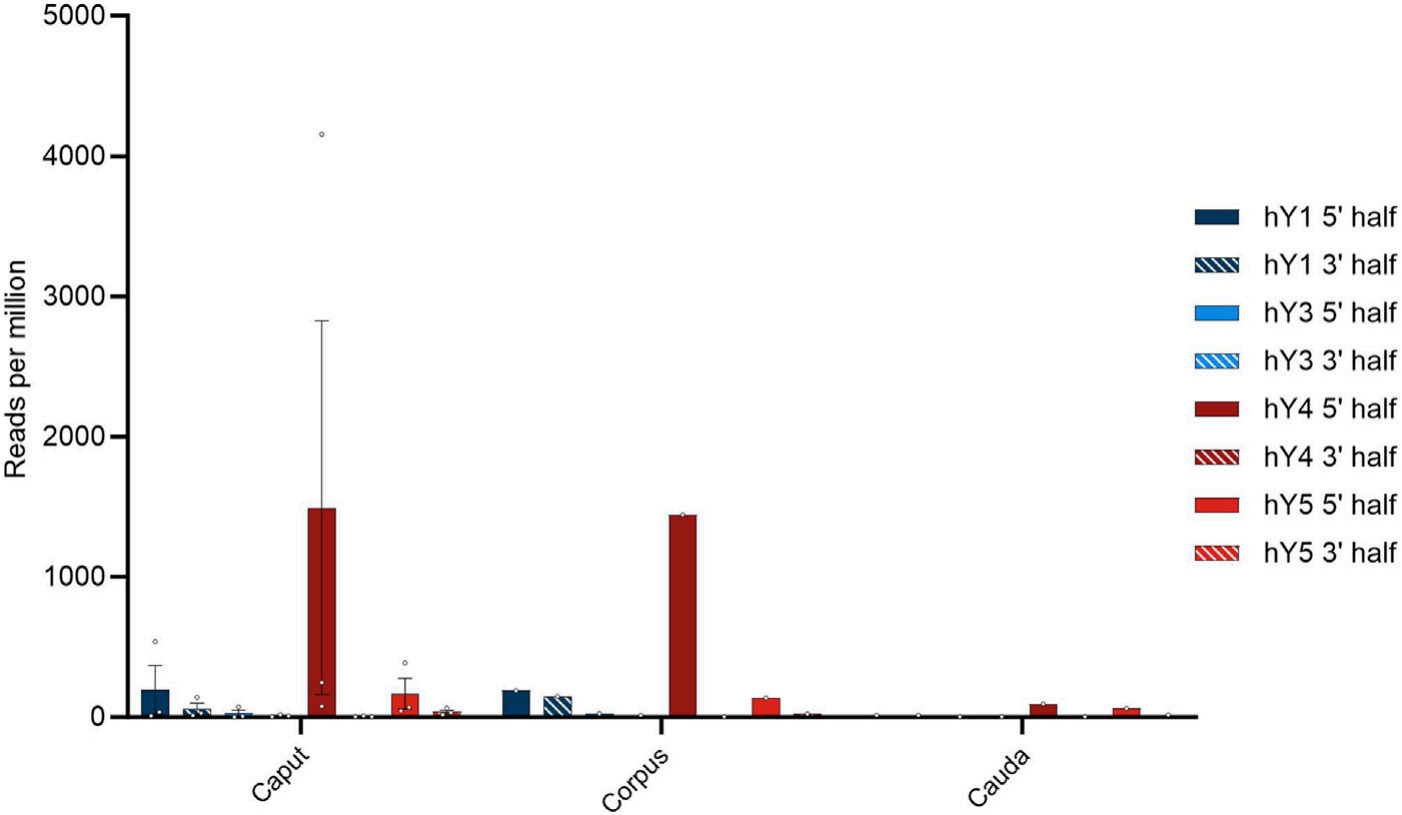
Profile of YsRNAs in the epididymis divided into caput, corpus, and cauda regions in reads per million alignable reads and depicted for the different YRNA homologs. Dots show the individual hits, and bars are depicted with error bars (SEM).

Next, we asked what role the reproductive support tissues possess in that respect. Spermatozoa mature during spermiohistogenesis and acquire functional competence. It is generally accepted that over a broad range of metazoans, the RNA payload in sperm is also controlled by the soma (Conine and Rando, 2022). Different reproductive support tissues are described for a taxonomically broad metazoan sample; for the mammalian site, a key player in this process is represented by the epididymis. To scrutinize if the YsRNAs are present in the epididymis, we downloaded RNA-Seq data obtained from Gong et al., (BioProject PRJNA821911). These data were divided into the epididymal regions of caput, corpus, and cauda. BLAST routines were carried out as above, and the results in hits per million hg38-alignable reads are depicted for the three epididymis regions and the YRNA sequences subdivided into the 5′ and 3′ regions (Figure 5).

The preponderance of Ys4RNA reflects both the situation in semen exosomes (see above) and in the epididymal samples. From this distribution of the abundances of individual YsRNA homologs, we conclude that the epididymis is a main determinant of the semen exosome profiles with respect to their YsRNA payload. The hits decrease with the passage through the epididymis ranging from high values in caput and corpus to smaller values in the caudal portion of the epididymis. This pattern largely recapitulates the observation of the soma-to-germline transfer via vesicles, as described by Conine and Rando (2022) in mice, albeit with the YsRNA as a typical payload in humans. Interestingly, the HENMT1 message, an enzyme responsible for the 3′ 2′-O-methylation, can be traced in the epididymis datasets, highlighting a possible role of the epididymis in the 3′ protection of YsRNAs that are transferred from soma to germline.

### 3.6 Sperm head Ro60 is not associated with YRNAs

YRNAs are bound by Ro60, a protein that also binds to misfolded non-coding RNAs, including pre-5S rRNA (Kowalski and Krude, 2015). In addition to other possible functions, Ro60 binds to endogenous Alu retroelements and regulates their expression. To test the functionality and substrate specificity of the Ro60 system in sperm heads, the profiles of Ro60-bound RNA were compared to the sperm head transcriptome (BioProject PRJNA890147), more precisely the non-coding RNA transcriptome including both small non-coding and long non-coding RNAs. For this purpose, RNA immunoprecipitation was performed and the bound RNAs were recorded in a transcriptome-wide readout. LincRNA transcriptome GTF files were obtained from the LNCipedia database (hg38) and used as input files for READCOUNT, following a mapping with HISAT2. The overall alignment rate was used to correct the total number of reads for the number of alignable reads in our dataset, and both transcriptomes and immunoprecipitation-obtained sequences were analyzed comparatively.

**FIGURE 6:**
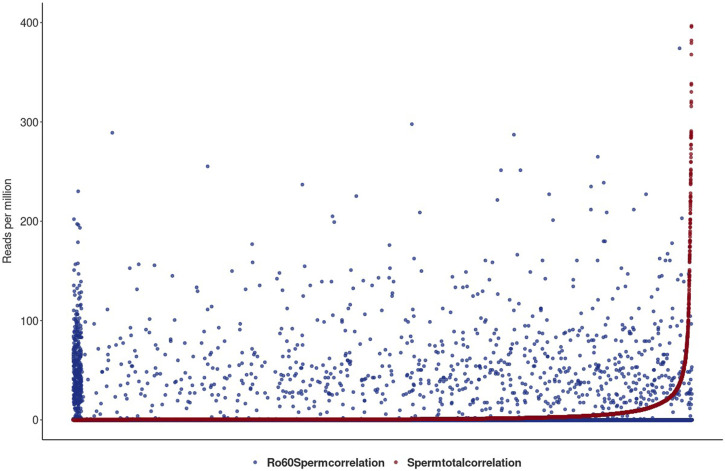
Illustration of the total sperm transcriptome–lincRNA profile in red and the Ro60-bound fraction in blue in reads per million alignable reads. On the x-axis, the lincRNA loci are listed. lincRNAs are sorted by the number of reads per million reads from the smallest to largest for the total sperm transcriptome. For better graphical readability, the y-axis was cut at 400 reads per million reads (many lincRNAs exceed this hit number in the total transcriptome).

Figure 6 shows the two profiles for the total lincRNA transcriptome in red and the Ro60-bound fraction in blue, respectively.

Figure 6 shows the lincRNA profile, as obtained from the total sperm transcriptome (red) together with the profile of the Ro60-bound lincRNA (blue) in reads per million alignable reads. Obviously, the profiles are markedly different and we conclude that Ro60 binding to lincRNA—as a prerequisite for chaperoning—is specific and apparently functional in sperm heads. Interestingly, and although we observed merely a small number of YRNA BLAST hits in the total transcriptome, Ro60 was not associated with YRNAs of the different homologous loci, as could be seen after querying the Ro60-RNA-ChIP-data with YRNA in a BLAST routine.

## 4 Discussion

In metazoans, the processes of gametogenesis, fertilization, and early embryonic development are characterized by erasure and restoration of epigenetic markers (Daxinger and Whitelaw, 2012). However, unlike in plants, where epigenetic inheritance is a widespread phenomenon (Henderson and Jacobsen, 2007), it is increasingly believed that at least some epigenetic information in metazoans can also be transmitted between generations. We focused on small non-coding RNAs as carriers of epigenetic information. Animal oocytes are much larger and carry more RNA than spermatozoa (Boerke et al., 2007), and the maternal load of long and small RNAs is essential for the initiation of embryogenesis (Schulz and Harrison, 2019). In contrast, the contribution of paternal RNAs to the zygote and their regulatory effects, especially prior to zygotic genome activation, are not fully understood. Our work initially focused on sperm heads to reflect the situation at the onset of male pronucleus formation and first performed an unbiased analysis of total small RNA cargo by a UNITAS annotation. Although the relative amounts of small RNAs annotated by UNITAS vary between individuals and biological replicates, clear trends can be observed, such as a relative lack of piRNAs in mature spermatocytes compared to the situation in the testis. In addition to mass abundance profiles of broad classes of small RNAs (between the testis and spermatozoa), we uncovered small YRNAs as a component of the small RNA profile in the sperm head.

YRNAs are short, non-coding RNAs that are transcribed from Pol III. In humans, there are four homologs encoded in a cluster on Chr. 7. The evolutionary history of this cluster includes gene losses and duplications during vertebrate evolution (Mosig et al., 2007). In addition, a substantial number of pseudogenes have been described for hg38 that coalesce relatively deeply. The transcripts of all YRNA homologs are approximately 100 nt long and are characterized by a 5′ triphosphorylation and a 3′ polyuridine tail that forms the La-binding site of the Pol III transcript. The secondary structures (Kowalski and Krude, 2015) show different stem-loop regions in all homologs, which can be assigned different functional roles. In particular, the lower stem domain is crucial for Ro60 binding and, thus, for the formation of RoRNPs. The YRNAs of the different homologs regulate the subcellular localization of Ro60 by binding to the outer surface of Ro60 and mainly regulate the entry of misfolded or defective non-coding RNAs into the circular Ro molecule. Smaller fragments from the YRNA source genes are referred to as YRNA-derived small RNAs (YsRNAs), which have been described for apoptotic cells, for example (Guglas et al., 2020). Because the sequencing strategy applied for small RNA includes size enrichment with a cut-off at 50 nt and SE50–NGS sequencing, we observed YsRNA in sperm heads with UNITAS, grouping them in miscRNA and to a lower extent in non-annotated sncRNAs, respectively.

To further discriminate between the different source YRNA homologs, we constructed a database using the homolog-specific sequences, as obtained from the NCBI to search for YRNA and YsRNA. To this end, the YRNA sequences were each divided in two halves, specifically to avoid confounding of our search results by the complementarity of the 5′ and 3′ ends. Local BLAST routines were carried out and only identical sequences counted as hits. From that, YsRNA profiles were obtained, which are generally characterized by the sheer absence of hits for the 3′ YsRNA halves and preponderance for Ys1RNA, followed by Ys4RNA and Ys3RNA in sperm heads. The least hit number was obtained for Ys5RNA. Next, we checked the YsRNA profiles in oocytes. To this end, we downloaded an SRA-deposited human oocyte single-cell dataset consisting of three biological replicates that were analyzed without further treatment and after an oxidation/ß-elimination experiment (BioProject PRJNA376426). This oocyte dataset was analyzed to unravel sncRNA patterns with an unprecedented resolution and sensitivity (Yang et al., 2019). The respective oocyte profiles were compared to the sperm head situation, which generated similar results with respect to the observation that 3’ halves of the YsRNA were underrepresented as well. However, the main observation was that Ys1RNA prevails in sperm heads, whereas it is virtually absent in oocytes. For the latter, we could detect Ys3RNAs as the major constituent of the YsRNA fractions.

To get a more precise picture of the YsRNAs, we separately counted the different lengths of the perfect hits with the result that the sperm head Ys1RNA peaks at 30 nt and 31 nt, falling into the size range of canonical piRNAs. These 30-nt and 31-nt Ys1RNAs are absent in human oocytes, thus representing a paternal contribution to the zygote introduced upon fertilization and upon forming the male pronucleus.

Since the Ys1RNAs fall in the size class of piRNAs and because the likely source YRNA is triphosphorylated at the 5′ end, we asked if we do find specific YsRNA modifications, with some of them having the potential to exclude the YsRNAs from successful adapter ligation and/or reverse transcription and thus excluding them from being represented in the NGS datasets. To check the 2′-O-methylation status at the 3′ terminus of YsRNA—a hallmark of piRNAs—before/after oxidation assays were performed in sperm heads. For this purpose, NaIO_4_ oxidation treatment and β-elimination protocols were carried out for the sperm heads' RNA, and the respective oxidation data from single-cell oocyte–small RNA seq (Yang et al., 2019) were compared. In addition, we check small RNA-sequenced human testis RNA in a before/after oxidation comparison for early stages of sperm development in a bulk testis RNA analysis. The most striking change inferred from UNITAS analysis of RNA is the enrichment of piRNA in sperm heads and concomitant reduction of most ncRNA classes, as annotated by UNITAS (with an exception of 5′ tRFs and lincRNA). Among YsRNAs in sperm heads, we observe a high abundance of 5′ halves of both Ys1RNAs and Ys4RNAs, both slightly decreasing after oxidation. In contrast, the much lower amounts of the respective YsRNA in oocytes completely disappear after oxidation/β-elimination. We conclude that Ys1RNAs and Ys4RNAs—besides possessing the canonical length—also exhibit the 2′-O-methylation at the 3′ terminus characteristic of canonical piRNAs and contrasting the situation in oocytes. We, therefore, propose that the sperm head YsRNA is a functional piRNA that is not encoded on piRNA clusters and thus not annotated as piRNAs by UNITAS. Querying the RNAcentral database (rnacentral.org) with the “search by sequence option,” the 30-nt and 31-nt-sized Ys1RNA fragment yielded piRNA hits in several species—not humans—including a new world monkey (*Callithrix jacchus*). Interestingly, this hit was obtained for analysis of *Callithrix* testis, whereas our human testis small RNA analysis exhibited a complete lack of YsRNAs in the testis, both in control and oxidation experiments.

Considering the 5′ triphosphate modifications of the source YRNA genes and possibly resolving cyclic 2′–3′ phosphates by the phosphatase activity of polynucleotide kinase (PNK), we set up an enzymatic treatment scheme with RppH and PNK treatment of total RNA obtained from sperm heads. Considering the RppH assays, we could not observe a great change in the abundance of the YsRNA, suggesting that in contrast to the triphosphorylated 5′ terminus of canonical full-length YRNAs, the respective fragments exhibited adapter-ligatable ends to be fully represented in our NGS output. In the PNK assay and more broadly, as determined by the UNITAS annotation, we found significant differences between the small RNA profiles before and after treatment. Most strikingly, PNK treatment increases the abundance of UNITAS-annotated rRNAs, suggesting a significant proportion of rRNA-annotated small RNAs with 2′,3′ -cyclic phosphate-containing 3′ ends. This observation partially confirms interpretations from Shigematsu et al. (2019), serving as an internal control of the PNK effect for the herein-presented data on YsRNA. With respect to YsRNAs, we did observe a prominent decrease in the abundance of YsRNAs after PNK treatment. Because RppH experiments indicate a monophosphate at the YsRNA 5′ terminus and 2′-O-methylation at the 3′ terminus prevents the formation of 2′,3′ -cyclic phosphate at the 3′ ends, we conclude that the 5′ phosphatase activity of PNK (Eun, H-M. 1996) catalyzes the removal of the canonical 5′ phosphates of the YsRNAs, thus hindering efficient ligation and representation in NGS libraries.

To detect the presence of YsRNAs in different stages of spermatogenesis, we checked SRA-deposited small RNA data from different stages and as obtained after microdissection of histologic sections from the human testis (Jan et al., 2017). In addition and in order to apply exactly the same NGS procedures as we did for sperm heads, human testicular bulk RNA was small RNA-sequenced. Corroborating the data from Jan et al. (2017) and since we covered sperm cells from all stages of spermatogenesis in our testicular RNA analysis and did not find YsRNAs, we conclude that YsRNAs are not present in the early stages of sperm development. It is known that spermatozoa gain full function only after leaving the testis and migrating through the epididymis, where they acquire motility and the ability to fertilize oocytes (Cornwall and von Horsten, 2007). Which role YsRNAs take in that process and how and when small RNAs, in general, are gained or lost during this post-testicular maturation is a question we attempted to answer.

Recent studies in mice suggest that sperms carry RNAs that are synthesized in epididymal somatic cells (Sharma et al., 2018). The data presented demonstrate that soma-germline RNA transfer occurs in male mammals, most likely via vesicular transport from the epididymis to maturing sperms. To elucidate if this is similarly realized for YsRNAs in humans as well, we queried NGS datasets of human semen exosomes and the human epididymis (BioProjects PRJNA242348 and PRJNA821911), the latter supposed to be a hub of soma-germline-transfer via vesicles in mice. Interestingly, we obtained rather similar YsRNA profiles upon comparing semen exosomes and the epididymis, subdivided into different structures, including caput, corpus, and cauda. The relative proportion of YsRNAs in the RNA preparations was very high, as compared to sperm heads with a preponderance of the Ys4RNA (5′ part) exceeding the amount of Ys1RNA (5′ part). The similarity between epididymal YsRNA profiles and semen exosome patterns suggests that these exosomes originated from the epididymis epithelium, most prominently from the caput and corpus regions. Our data extend the observations of a high dynamic of the small RNA payload during spermatogenesis in the murine model system (Sharma et al., 2018) by the formation of YsRNAs and soma to germline transfer during spermiohistogenesis in the epididymis in humans. Although, mechanistically, several questions are still open, upon checking the presence of HENMT1—the factor that is responsible for catalyzing the 2′-O-methylation at the 3′ end of piRNAs—we could trace the respective message in the epididymal transcriptomes. The epididymis is thus a highly likely tissue to carry out essential steps of YsRNA biogenesis in humans, rendering the source molecule of YRNAs into a canonical piRNA that is later transferred between soma and germline. This epigenetic inheritance in humans represents a paternal contribution to the zygote and, more importantly, is not restricted to intergenerational inheritance but represents the hallmarks of transgenerational inheritance, where the soma-germline transfer occurs in every generation. Since the soma–germline transfer of individual classes of sncRNAs seems to vary among species, there is an obvious quest for the adaptive value of the paternal contribution of YsRNAs to the zygote upon fertilization in humans. Human sperms require an epididymal passage to become a functional sperm cell. It is widely recognized and has been impressively demonstrated by Liu et al. (2001) that sperm origin is critical for intracytoplasmic sperm injection (ICSI)—the most commonly used technique to aid reproduction in humans. Using epididymal sperms appears to adversely affect the morphologic grade and cleavage stage of the resulting embryos, as compared to the ejaculated sperm. Overall, this leads to a differential reproduction or—synonymously—a selection phenomenon. Although it is difficult to link this general phenomenon to the mechanisms by which these YsRNAs may achieve regulation in progeny, we hypothesize that—in the case of YsRNAs—this may be related to the function of the source YRNAs, which act as a gatekeeper for the RNA-chaperoning Ro60 system.

At first, we detected Ro60-bound RNA in the sperm head with a binding profile that we interpret as functional and specific when compared to the total RNA payload in human sperm heads. Ro60 protein is, thus, present in sperm heads and was recently shown to be present in human oocytes, as determined by mass spectrometry (Dang et al., 2023). Upon fertilization and before the zygotic genome activation is realized, a process that requires Pol III transcripts to ensure proper translation, functional, and 2′-O-methyl-protected YsRNA is delivered into the zygote via the male pronucleus. Upon Pol III transcription, we hypothesize that YRNAs are synthesized abundantly which could—upon binding to Ro60—significantly reduce the RNA-chaperoning activity of Ro60. With this in mind, we speculate that the presence of paternally contributed YsRNAs in a molar ratio exceeding the oocyte and early embryo YRNA might interfere with the folding of the YRNA stem domain that is binding to Ro60, resulting in a more efficient RNA-chaperoning activity of Ro60 in early embryogenesis.

Because our hypothesis is mainly correlative, further experiments are warranted to understand the largely enigmatic role of the sperm RNA payload in the early embryo and the associated regulatory phenomena. On one side, our results contribute to knowledge about the importance of epigenetic inheritance in recent human evolution and its possible adaptive value. Second, the knowledge of the biogenesis and profile of small RNAs as epigenetic information carriers in human mature sperms could be of medical importance as well, especially for reproduction-assisting techniques. Mammalian model systems might offer the opportunity to interfere with the soma-germline transfer, the herein-involved vesicles, and their small RNA cargo, to directly observe the quantitative effects on reproduction. With the use of small extracellular vesicles as “delivery vesicles” in oncology in mind, it is reasonable to assume that assisted reproduction techniques could benefit from the findings on small RNA as epigenetic information carriers in human sperms transmitted between soma and germline.

## Data Availability

The datasets presented in this study can be found in online repositories. The names of the repository/repositories and accession number(s) can be found at: https://www.ncbi.nlm.nih.gov/, PRJNA1016274.
