# Supplementary material for: Human sperm heads harbor modified YsRNA as transgenerationally inherited non-coding RNAs
Source: Front Genet. 2023 Dec 13;14:1294389. doi: 10.3389/fgene.2023.1294389 (PMC10756665; doi:10.3389/fgene.2023.1294389)
Supplement: Supplementary file 2 [file Image2.pdf]

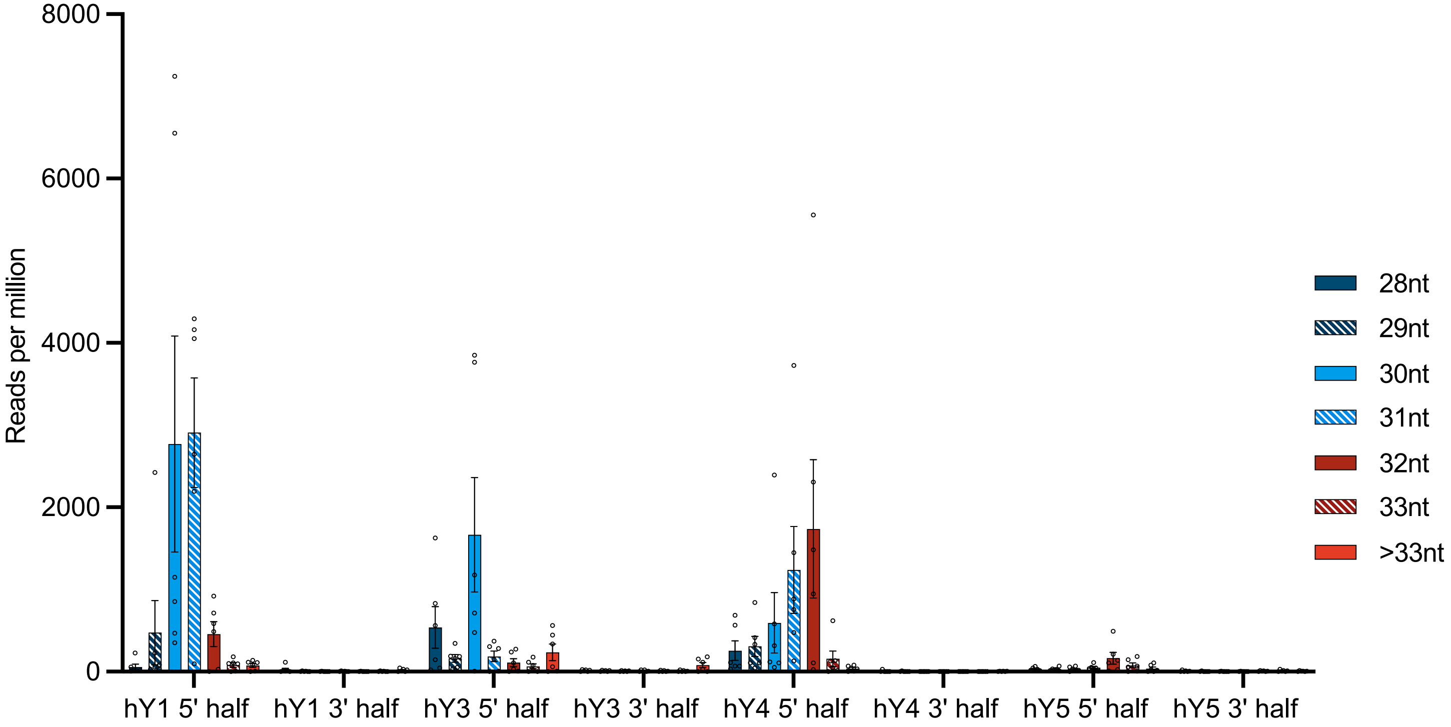

Supplementary Figure 2: Hit numbers for the 5’ and 3’ parts of the four YRNA homologs in sperm heads are shown in mean values and given in reads per million alignable reads. Lengths of the YsRNA are given in different collors as indicated on the right. Dots indicate individual hit numbers and bars show the standard error of mean (SEM).
