# Supplementary material for: Human sperm heads harbor modified YsRNA as transgenerationally inherited non-coding RNAs
Source: Front Genet. 2023 Dec 13;14:1294389. doi: 10.3389/fgene.2023.1294389 (PMC10756665; doi:10.3389/fgene.2023.1294389)
Supplement: Supplementary file 5 [file Image1.pdf]

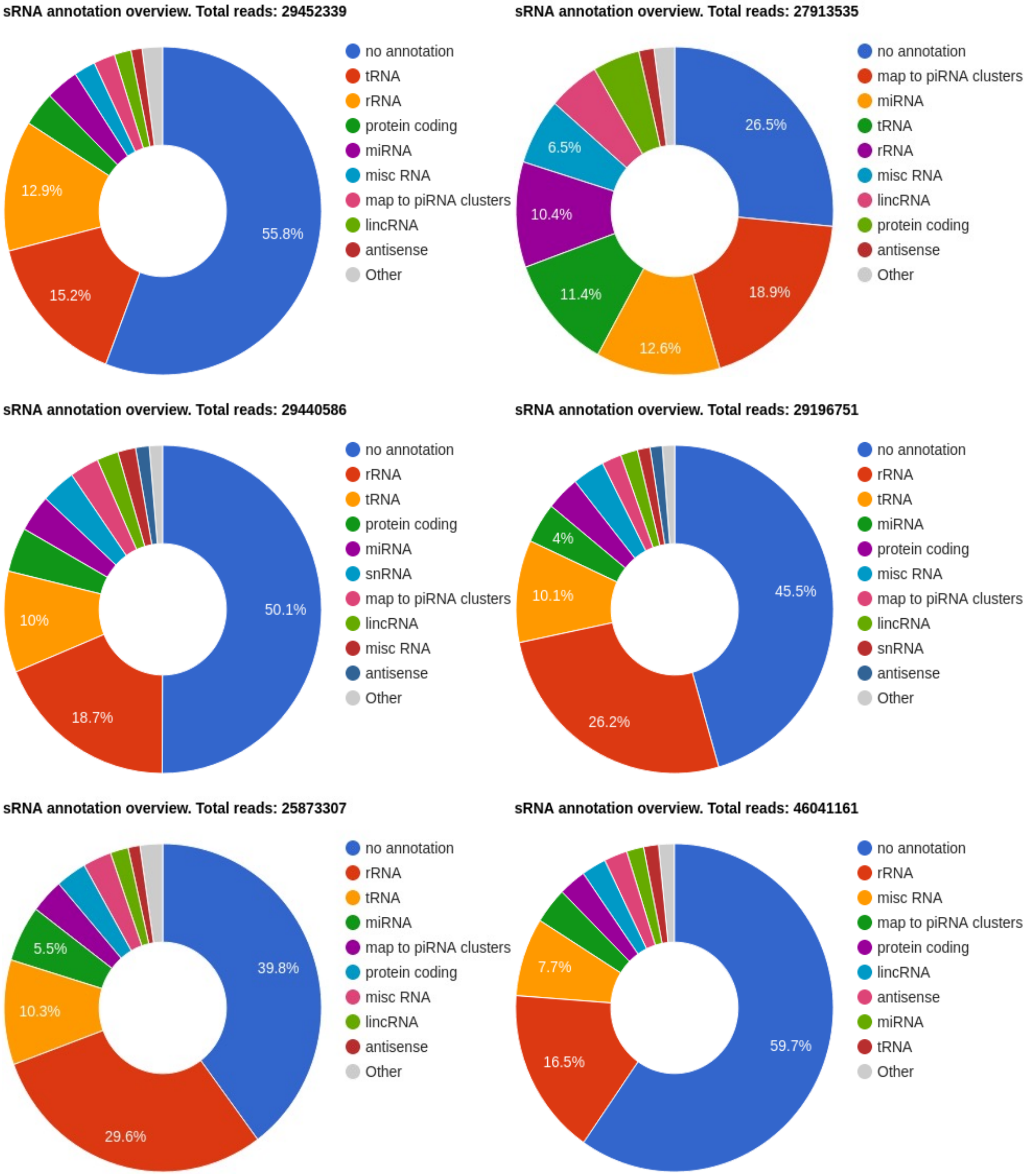

Supplementary Figure 1: Donut chart of the UNITAS annotation for the six sperm head samples.
